# The Resveratrol Trimer Miyabenol C Inhibits β-Secretase Activity and β-Amyloid Generation

**DOI:** 10.1371/journal.pone.0115973

**Published:** 2015-01-28

**Authors:** Jin Hu, Ting Lin, Yuehong Gao, Junyue Xu, Chao Jiang, Guanghui Wang, Guojun Bu, Huaxi Xu, Haifeng Chen, Yun-wu Zhang

**Affiliations:** 1 Fujian Provincial Key Laboratory of Neurodegenerative Disease and Aging Research, School of Pharmaceutical Sciences, Xiamen University, Xiamen, 361102, China; 2 Institute of Neuroscience, College of Medicine, Xiamen University, Xiamen, 361102, China; Nathan Kline Institute and New York University Langone Medical Center, UNITED STATES

## Abstract

Accumulation and deposition of amyloid-β peptide (Aβ) in the brain is a primary cause of the pathogenesis of Alzheimer’s disease (AD). Aβ is generated from amyloid-β precursor protein (APP) through sequential cleavages first by β-secretase and then by γ-secretase. Inhibiting β-secretase activity is believed to be one of the most promising strategies for AD treatment. In the present study, we found that a resveratrol trimer, miyabenol C, isolated from stems and leaves of the small-leaf grape (*Vitisthunbergii var*. taiwaniana), can markedly reduce Aβ and sAPPβ levels in both cell cultures and the brain of AD model mice. Mechanistic studies revealed that miyabenol C affects neither protein levels of APP, the two major α-secretases ADAM10 and TACE, and the γ-secretase component Presenilin 1, nor γ-secretase-mediated Notch processing and TACE activity. In contrast, although miyabenol C has no effect on altering protein levels of the β-secretase BACE1, it can inhibit both *in vitro* and *in vivo* β-secretase activity. Together, our results indicate that miyabenol C is a prominent β-secretase inhibitor and lead compound for AD drug development.

## Introduction

Alzheimer’s disease (AD) is a devastating neurodegenerative disorder characterized by impaired memory and cognition. One of the major pathological hallmarks of AD in the brain is senile plaques, which are composed of heterozygous amyloid-β (Aβ) peptides. Ample evidence indicates that accumulation of Aβ peptides in vulnerable brain regions plays a central role in AD pathogenesis: Aβ is neurotoxic and can trigger a cascade of neurodegenerative steps including the formation of senile plaques and neurofibrillary tangles, synaptic dysfunction, and eventual neuronal loss [1, 2]. Aβ is a proteolytic product of the amyloid-β precursor protein (APP) and is generated through sequential cleavages by enzymes called β- and γ-secretases. During this amyloidogenic processing, β-secretase first cleaves the type I transmembrane APP protein to generate an extracellular fragment known as sAPPβ and a membrane-associated carboxyl-terminal fragment known as APP β-CTF. APP β-CTF is then cleaved by γ-secretase to release Aβ. Alternatively, APP can be subjected to a non-amyloidogenic processing and cleaved by α-secretase within the Aβ domain. α-secretase-mediated cleavage precludes Aβ generation and generates an extracellular domain of APP known as sAPPα instead [3, 4].

β-cleavage of APP is the first and rate-limiting step in Aβ production. The transmembranous aspartic protease β-site APP cleaving enzyme 1 (BACE1) has been identified as the essential β-secretase *in vivo*[5–8]. The level and activity of BACE1 are found to be elevated in postmortem brain of sporadic AD patients [9–11], suggesting a causative role of BACE1 in AD. Although homozygous BACE1 knock-out mice develop certain phenotypic abnormalities including reduced body size, hyperactive behavior, decreased grip strength and elevated pain sensitivity [12–14], probably because the cleavage of other BACE1 substrates such as neuregulin 1 [13, 14] and β-subunits of voltage-gated sodium channels [15] is blocked, BACE1 heterozygous knockout mice do not develop such abnormal phenotypes and heterozygous knockout of BACE1 still can reduce Aβ deposition in AD mice [16, 17]. Therefore, moderate inhibition of β-secretase activity is considered as a promising strategy for AD intervention.

Natural products have been recognized as sources of new lead compounds for the treatment of various diseases including AD [18, 19]. The small-leaf grape *Vitisthunbergii var*. taiwaniana (VTT) is a wild grape native to Taiwan where, along with other species of *Vitis spp*., is used as a folk medicine [20]. The extracts or purified compounds from VTT have been reported to have anti-microbial [21], anti-inflammatory [22], anti-hypertensive [23] and neuroprotective activities [20]. In the present study, we isolated a resveratrol trimer, miyabenol C, from the stem and leaf extracts of VTT. Importantly, we demonstrate that miyabenol C is a prominent β-secretase inhibitor and can reduce Aβ levels both *in vitro* and *in vivo*, suggesting that miyabenol C may be a lead compound for AD drug development.

## Materials and Methods

### Isolation of miyabenol C from stem and leaf extracts of VTT

Dried stems and leaves of VTT (5.5 kg) were boiled and refluxed for 2.5 h with 60% of aqueous ethanol solution (10 L × 2 times). After filtration, the extracted solution was concentrated *in vacuo*. The extract (230.3 g) was then separated over a Diaion HP-20 column, using EtOH-H_2_O as mobile phase, into three fractions (A-C). Fraction B (60% EtOH-H_2_O eluent, 130.0 g) was chromatographed on silica gel column using stepwise gradient elution with CHCl_3_-MeOH (100:0∼0:100) to obtain 11 fractions (Fr. 1-11). Fr.5 (5.0 g) was subjected to silica gel column chromatography and eluted with CHCl_3_-MeOH (95:5, 9:1, 85:15, 0:100) to generate 8 sub-fractions (Fr.5-1 ∼ Fr.5-8). Fr. 5-5 (835 mg) was applied to ODS column chromatography and eluted with MeOH-H_2_O (3:7, 4:6 and 1:1). The elution of MeOH-H_2_O (4:6) was further repeated by RP-18 and purified through preparative HPLC (RestekPrinnacle DB C_18_, 5 μm, 250×20 mm) with elution of 45% aqueous methanol solution to generate a pure compound (65mg), whose structure was then determined by means of NMR and MS.

### Cell culture and treatment

Maintenance of human neuroblastoma SH-SY5Y cells [24], mouse neuroblastoma N2a naive cells (N2aWT) [25] and N2a cells stably expressing human APP695 (N2a695) [26] was as previously reported [27, 28].

Purified miyabenol C and β-secretase inhibitor II (Millipore) were dissolved in DMSO (Sigma) as 20 mM stock solution. For treatment, cells grown to confluency were switched to be incubated in FBS-free media that were added with indicated concentrations of miyabenol C, β-secretase inhibitor II or DMSO for 10h.

### Cell viability test

Cell viability was measured using the Cell Counting Kit-8 (CCK-8) assay (Dojindo), following the manufacturer’s protocol.

### Western blot and antibodies

Conditioned media from treated cells were assayed for sAPPα and sAPPβ by Western blot. Treated cells were lysed in ice-cold lysis buffer (10mM Tris pH7.5, 150mM NaCl, 1mM EDTA, 1% NP-40, 1× Protease Inhibitor Cocktail), and equal protein amounts of cell lysates were analyzed by Western blot. Antibodies used for Western blot included: 369 (against the carboxyl terminus of APP, 1:1000) and ab14 (against PS1 amino-terminal fragment, 1:1000) developed in our laboratory; 6E10 (against sAPPα and β-CTF, 1:1000) and anti-sAPPβ antibodies (1:500) from Covance; anti-α-tubulin (1:10000) and anti-ADAM10 (1:1000) antibodies from Millipore; anti-ADAM17 (1:1000) antibody from Abcam; and anti-c-Myc (1:1000) and anti-NICD (1:1000) antibodies from CST. The mouse monoclonal antibody 3D5 (against BACE1, 1:1000) was a kind gift from Dr. Robert Vassar at Northwestern University.

### NotchΔE cDNA construct and cell based γ-secretase activity assay

NotchΔE fragment contains the transmembrane and intracellular domains of Notch1 and is an immediate substrate of γ-secretase for generating Notch intracellular domain (NICD). For γ-secretase activity assay, N2aWT cells were transiently transfected with the NotchΔE plasmid. After splitting equally, cells were treated with DMSO, the γ-secretase inhibitor compound E (Millipore) or miyabenol C for 10h. Cell lysates were analyzed by Western blot for NotchΔE and NICD levels. Cellular γ-secretase activity was estimated by the generation of NICD.

### α-secretase activity assay

The activity of α-secretase in cells was measured by using InnoZyme *TACE* Activity Kit (Millipore), following the manufacturer’s protocols.

### β-secretase activity assays

Cell-based β-secretase activity and cell-free BACE1 activity were measured by using commercial Kits from Millipore and Sigma, respectively, following the manufacturers’ protocols.

### Aβ_40_ and Aβ_42_ ELISA assays

Human Aβ_40_ and Aβ_42_ in conditioned media from treated N2a695 cells were assayed by sandwich ELISA, following a previously described protocol [29].

### Ethics statement

All animal procedures were in accordance with the National Institute of Health Guidelines for the Care and Use of Laboratory Animals and were approved by the Animal Ethics Committee of Xiamen University (IACUC #: XMULAC20120012).

### Miyabenol C treatment of APP/PS1 mice and sample analysis

C57BL6 mice co-expressing the Swedish mutant APP and the exon-9 deletion mutant PS1 (APP/PS1) were provided by Nanjing Biomedical Research Institute of Nanjing University, China. For treatment, 12-month-old APP/PS1 transgenic mice were anesthetized with sodium pentobarbital (50μg/g) and placed in a stereotaxic apparatus before intracerebroventricular injection of vehicle (45% DMSO in artificial cerebrospinal fluid: 148 mM NaCl, 3 mM KCl, 1.4 mM CaCl_2_, 0.75 mM MgCl_2_, 0.8 mM Na_2_HPO_4_, 0.2 mM NaH_2_PO_4_) or miyabenol C (0.6μg/g). Vehicle and miyabenol C solution were injected at 4μL into the lateral ventricle using a 5μL-blunt needle equipped with asyringe pump (KD Scientific). The stereotaxic coordinates for the lateral ventricle were AP 0.5 (0.5 mm posterior to bregma), L 1 (1 mm left from mid-sagittal line) and H 2.2 (2.2 mm below bregma). Sixty seconds after insertion of the needle, vehicle or miyabenol C solution were injected at a constant flow rate of 0.4μL/min. The injection needle was kept in place for an additional 10 min to prevent reflux of fluid.

Three days after treatment, mice were anesthetized and euthanized by transcardial perfusion with ice-cold physiological saline. Brain cortex and hippocampus were dissected and lysed for Western blot analysis of APP and sAPPβ. Alternatively, samples were weighed and sequentially extracted into TBSX- (25 mM Tris-HCl, pH 7.4, 150 mM NaCl, 1% Triton X-100) soluble and GuHCl-soluble fractions for Aβ sandwich ELISAs. This separation was carried out following a previously validated method [30] with some modification. Briefly, samples were placed in 2mL ice-cold glass dounce homogenizer containing TBSX homogenization buffer. Samples were homogenized on ice, transferred into pre-chilled 1.5 mL polyallomer ultracentrifuge tubes, and centrifuged at 100,000×g for 1 h at 4°C. The supernatant was collected as TBSX-soluble fraction. The pellet was re-suspended in 5M GuHCl, mixed by rotation at room temperature for 6 h, and centrifuged at 16,000×g for 30 min. The supernatant was collected as GuHCl soluble fraction.

### Statistical analysis

The statistical analysis was carried out by using GraphPad Prism Software 5.0. Data were analyzed by Student’s t-test or One-way analysis of variance (ANOVA) followed by Tukey’s post-hoc test, and presented as the mean ± standard deviation.

## Results

### Isolation and identification of miyabenol C

**Figure 1:**
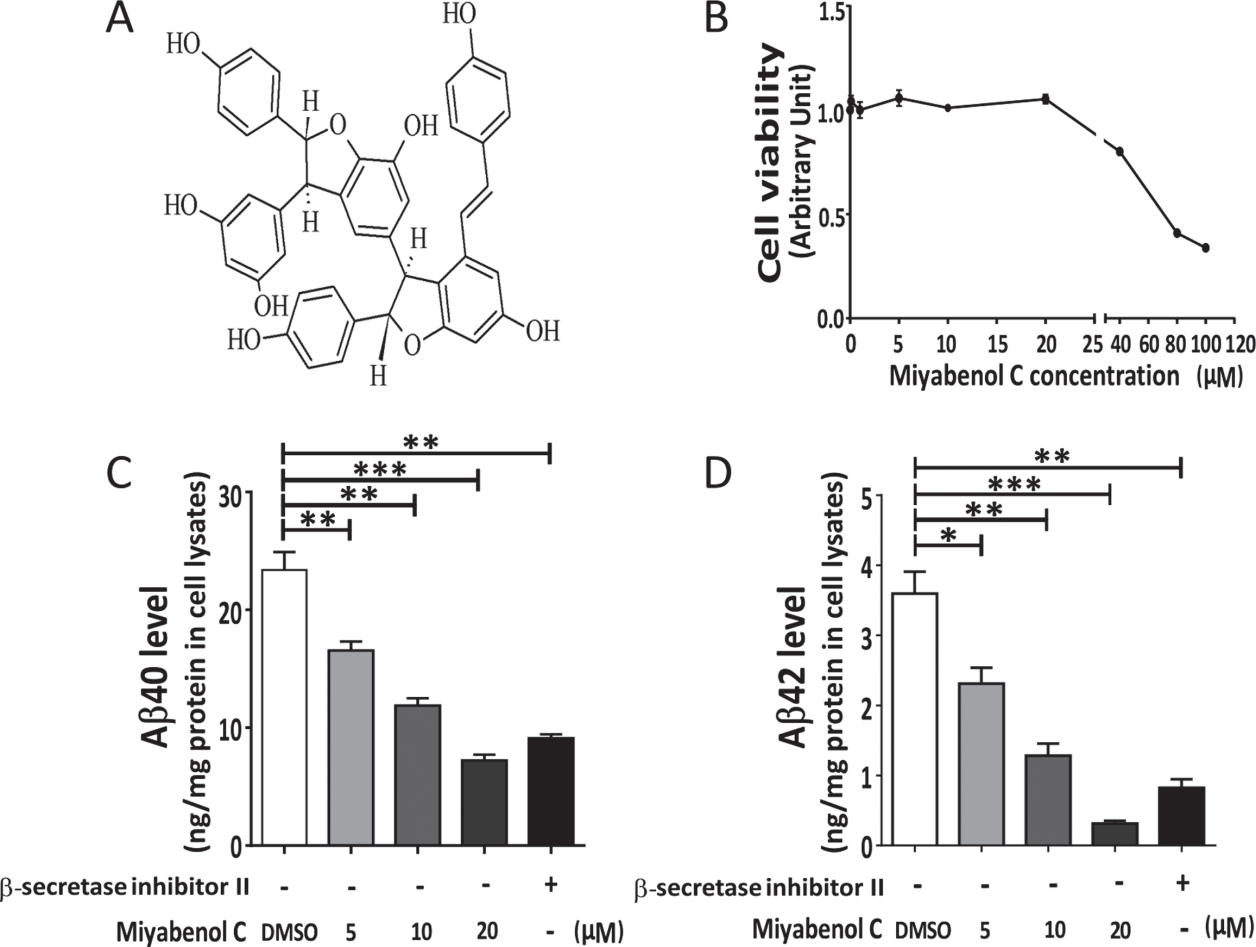
Miyabenol C treatment inhibits Aβ secretion. (A) Chemical structure of miyabenol C. (B) N2a695 cells were treated with indicated doses of miyabenol C for 10h. Cell viability was measured using CCK-8 assay, n = 3. Error bars indicate standard deviation. (C, D) N2a695 cells were treated with DMSO (negative control), β-secretase inhibitor II (positive control, 2μM) or indicated doses of miyabenol C for 10h. Extracellular Aβ40 (C) and Aβ42 (D) levels were quantified by ELISA (One-way ANOVA followed by Tukey’s post hoc test, n = 3, *: *p*< 0.05, **: *p*< 0.01, ***: *p*< 0.001).

We isolated a series of compounds from stems and leaves of VTT (unpublished data). One of the compounds was purified in a yellow and solid form. This compound was subjected to MS and NMR and the spectral data were:

ESI-MS (Bruker FTMS): m/z 679 [M-H]^−^. ^1^H-NMR (BrukerAvance spectrometer, DMSO-d6, 400MHz) δ: 7.18 (2H, d, *J* = 8.8Hz, H-2a, H-6a), 7.13 (2H, d, *J* = 8.4Hz, H-2c, H-6c), 6.90 (1H, d, *J* = 16.4Hz, H-7c), 6.84 (2H, d, *J* = 8.8Hz, H-3a, H-5a), 6.75 (2H, d, *J* = 8.4Hz, H-3c, H-5c), 6.62 (1H, d, *J* = 16.4Hz, H-8c), 6.58 (2H, d, *J* = 8.8Hz, H-3b, H-5b), 6.52 (2H, d, *J* = 8.8Hz, H-2b, H-6b), 6.32 I (1H, d, *J* = 2.0Hz, H-12b), 6.24 (1H, d, *J* = 2.0Hz, H-12a), 6.19 (2H, d, *J* = 2.0Hz, H-10a, H-14a), 6.09 (1H, d, *J* = 2.0Hz, H-14b), 5.39 (1H, d, *J* = 5.2Hz, H-7a), 5.22 (1H, d, *J* = 1.2Hz, H-7b), 4.64 (1H, d, *J* = 5.2Hz, H-8a), 4.33 (1H, d, *J* = 1.2Hz, H-8b). 13C-NMR (BrukerAvance spectrometer, DMSO-d6,100MHz) δ: 162.3 (C-11b), 160.7 (H-11c), 160.3 (C-13b), 160.3 (C-13a), 160.3 (C-11a), 159.5 (C-13c), 158.2 (C-4c), 157.6 (C-4b), 157.0 (C-4a), 147.6 (C-9a), 143.5 (C-9b), 135.9 (C-9c), 133.4(C-1a), 133.3 (C-1b), 131.2 (C-7c), 129.3 (C-1c), 128.7 (C-2c, C-6c), 127.8 ( C-2a, C-6a), 127.4 (C-2b, C-6b), 122.8 (C-8c), 121.4 (C-10c), 118.6 (C-10b), 116.7 (C-3c, C-5c), 116.5 (C-3a, C-5a), 115.7 (C-3b, C-5b), 107.7 (C-14b), 107 (C-10a, C-14a), 104.4 (C-14c), 102.6 (C-12a), 97.2 (C-12c), 96.4 (C-12b), 94.6 (C-7a), 92.3 (C-7b), 57.2 (C-8a), 51.1 (C-8b). By comparison, we found that the spectral data were identical to that of miyabenol C (Fig. 1A), a previously reported resveratrol trimer [31], indicating that the compound we isolated is miyabenol C.

### Miyabenol C treatment reduces Aβ secretion

To study whether miyabenol C can affect Aβ generation, we first measured its cytotoxicity. N2a695 cells were treated with increasing doses of miyabenol C (0, 0.1, 1, 5, 10, 20, 40, 80, 100μM) for 10h and cell viability was evaluated by CCK-8 assay. The results showed that miyabenol C had no cytotoxicity at lower doses (5–20μM) but showed a dose-dependent cytotoxicity at higher doses (40–100μM) (Fig. 1B). Therefore, we used doses of 0–20μM in the following studies. When N2a695 cells were treated with miyabenol C (5, 10, 20μM) for 10 h, levels of Aβ40 (Fig. 1C) and Aβ42 (Fig. 1D) in conditioned media were markedly decreased in a dose-dependent manner.

### Miyabenol C does not affect γ-secretase activity

**Figure 2:**
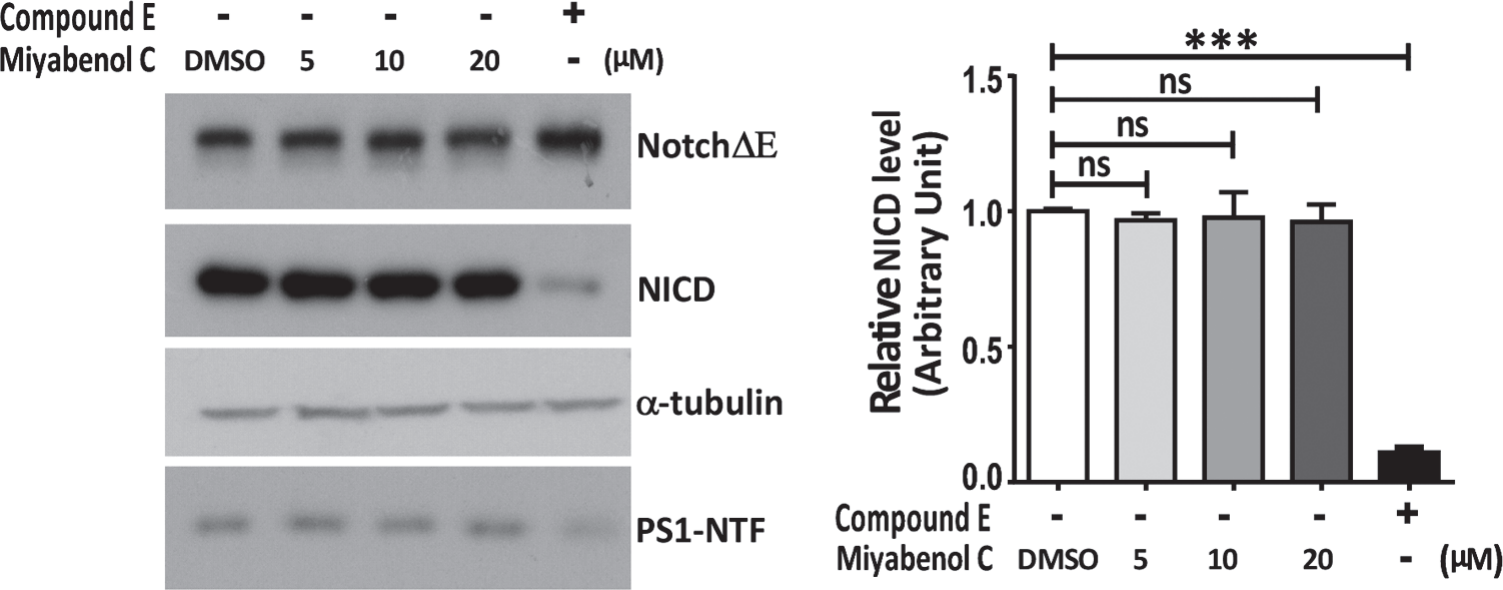
Miyabenol C does not inhibit γ-secretase activity. N2aWT cells were first transfected with NotchΔE. After splitting equally, cells were treated with DMSO (negative control), the γ-secretase inhibitor compound E (positive control, 0.5μM) or indicated doses of miyabenol C for 10h. Equal protein amounts of cell lysates were subjected to SDS-PAGE and Western blot. NICD levels were quantified by densitometry using image J for comparison (One-way ANOVA followed by Tukey’s post hoc test, n = 3, ns: *p*> 0.05, ***: *p*< 0.001).

Because γ-secretase-mediated cleavage of APP is the final step for Aβ generation, it is possible that miyabenol C may inhibit γ-secretase activity and reduce Aβ production. To study this possibility, we transfected N2aWT cells with NotchΔE that can be directly cleaved by γ-secretase for NICD generation [32, 33]. Cells were then treated with miyabenol C. The generation of NICD was measured by Western blot and the results showed that miyabenol C had no effect on inhibiting NICD generation, whereas the γ-secretase inhibitor compound E dramatically blocked NICD generation (Fig. 2). In addition, miyabenol C treatment did not affect the protein levels of PS1, a major γ-secretase component (Fig. 2). These results suggest that miyabenol C does not inhibit γ-secretase activity.

### Miyabenol C inhibits APP β-cleavage and promotes APP α-cleavage

**Figure 3:**
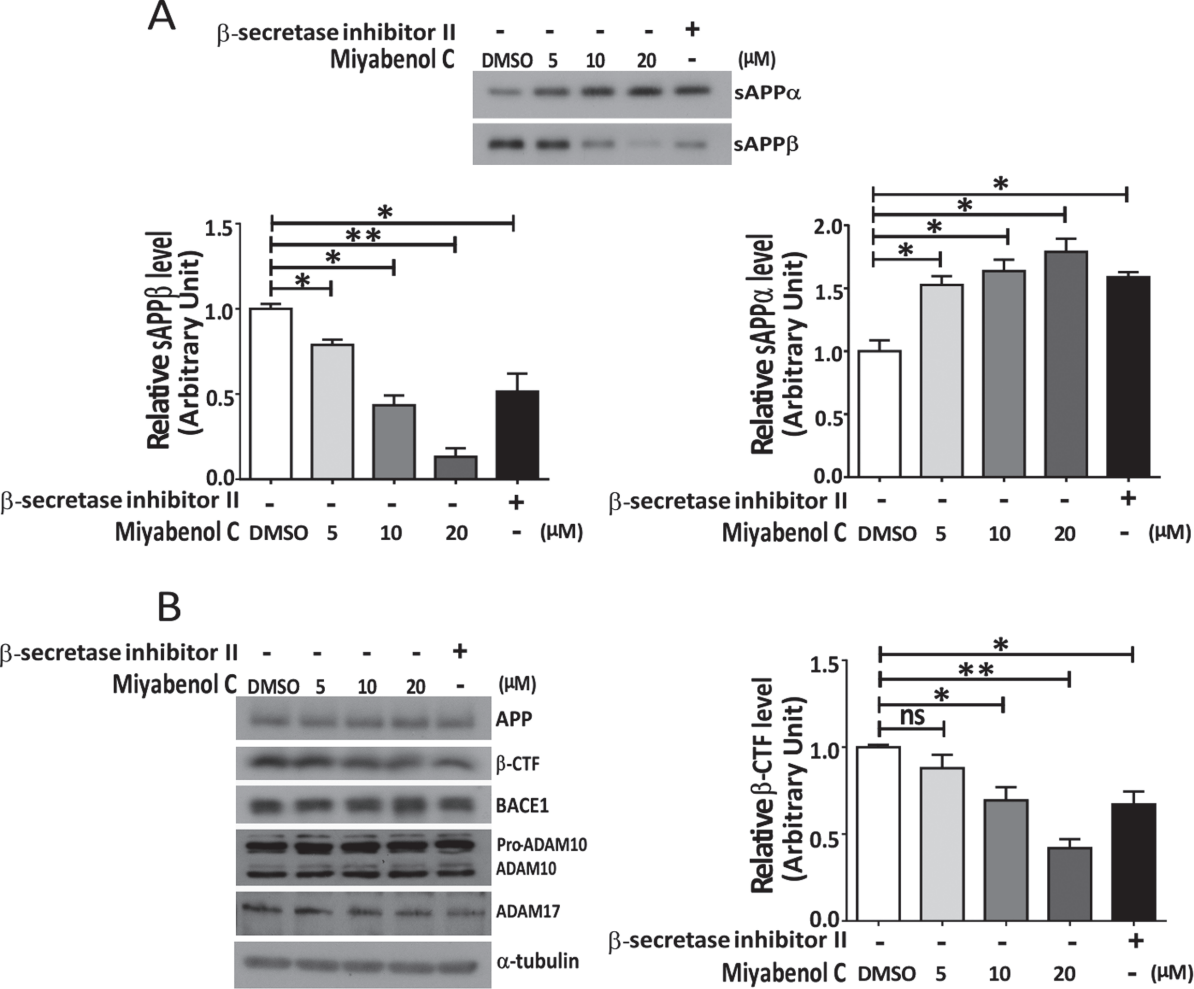
Miyabenol C treatment reduces APP β-CTF and sAPPβ levels and increases sAPPα levels. N2a695 cells were treated with DMSO (negative control), β-secretase inhibitor II (2μM) or indicated doses of miyabenol C for 10h. (A) Conditioned media and (B) cellular lysates were subjected to SDS-PAGE and Western blot. sAPPα, sAPPβ and β-CTF levels were quantified by densitometry using image J for comparison (One-way ANOVA followed by Tukey’s post hoc test, n = 3, ns: *p*>0.05, *: *p*<0.05, **: *p*<0.01).

Because β-secretase-mediated APP processing is the first step leading to Aβ generation, we studied whether miyabenol C affects β-secretase. In N2a695 cells, miyabenol C treatment dose-dependently decreased the secreted level of sAPPβ, an amino-terminal fragment of APP generated by β-secretase cleavage (Fig. 3A). Consistently, the level of APP β-CTF (a carboxyl-terminal fragment of APP generated by β-secretase cleavage) was also decreased upon miyabenol C treatment (Fig. 3B). These results suggest that miyabenol C inhibits β-cleavage of APP. In addition, miyabenol C treatments increased the level of secreted sAPPα, the major extracellular fragment of APP released by α-secretase cleavage (Fig. 3A). Moreover, we found that miyabenol C treatment did not affect protein levels of APP, the major β-secretase BACE1 and two major α-secretases ADAM10 and ADAM17 (i.e. Tumor necrosis factor alpha converting enzyme, TACE) [34–36] (Fig. 3B). These results suggest that miyabenol C reduces APP amyloidogenic processing not through affecting α- and β-secretase protein levels.

### Miyabenol C inhibits β- but not α-secretase activity

**Figure 4:**
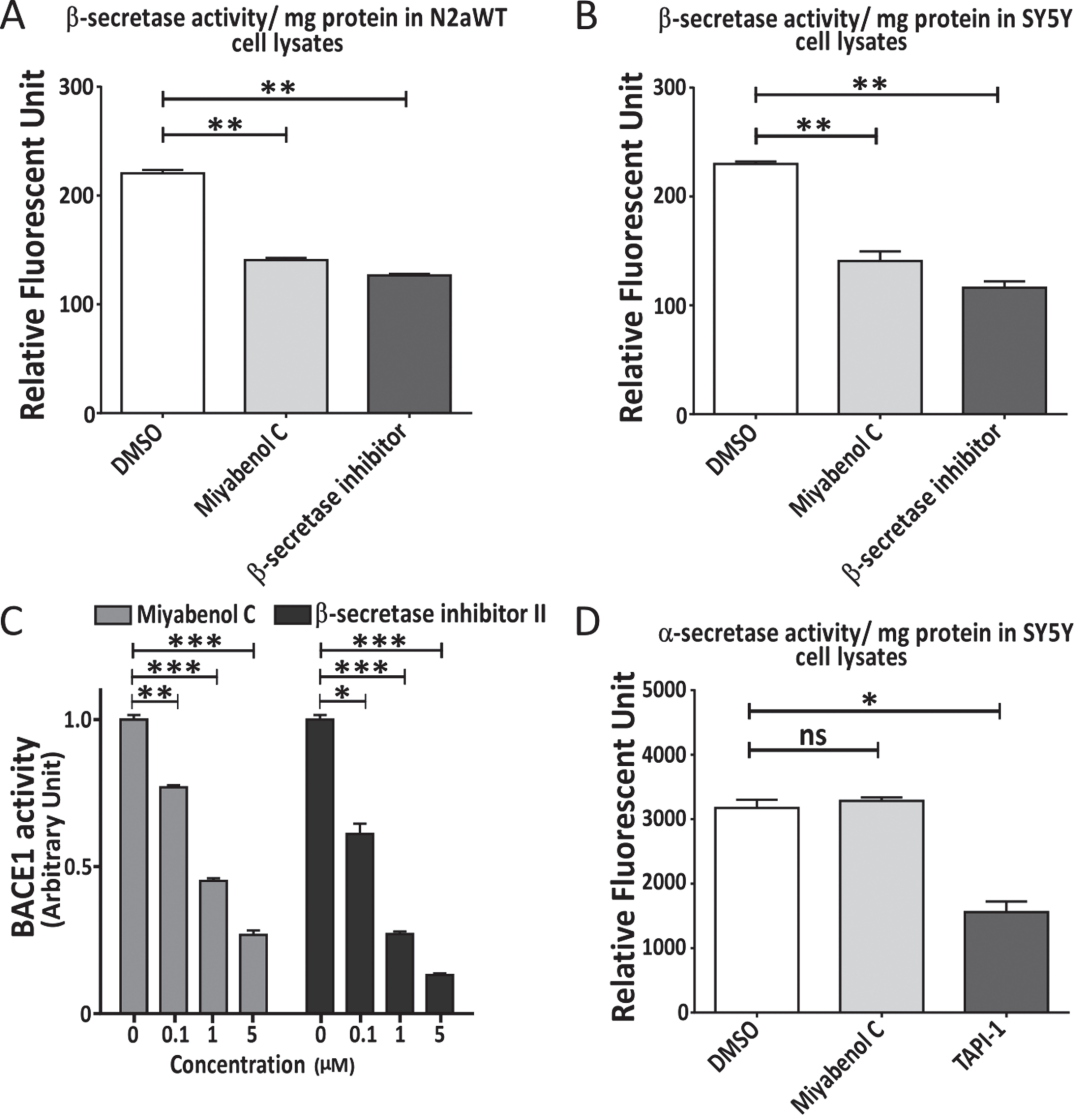
Miyabenol C inhibits β-secretase activity. (A) N2aWT and (B) SH-SY5Y cells were treated with DMSO (negative control), miyabenol C (10μM) or a β-secretase inhibitor (positive control, 2μM, provided in the kit) for 10h. Cell lysates were assayed for β-secretase activity by using a commercial kit from Millipore and subjected to comparison. (C) Indicated amounts of miyabenol C and β-secretase inhibitor II were incubated with BACE1 and its substrate provided by a commercial kit from Sigma, for 2h at 37°C. BACE1 activity was measured for comparison. (D) SY5Y cells were treated with DMSO (negative control), miyabenol C (10μM) or the α-secretase inhibitor TAPI-1 (positive control, 10μM) for 10h. Cell lysates were assayed for the α-secretase (TACE) activity for comparison (One-way ANOVA followed by Tukey’s post hoc test, n = 3, ns: *p*> 0.05, *: *p*< 0.05, **: *p*< 0.01, ***: *p*< 0.001).

Since miyabenol C inhibits β-processing of APP without affecting BACE1 levels, miyabenol C probably directly inhibits BACE1 activity. To ascertain this possibility, we carried out a cell-based assay to measure the β-secretase activity. The results revealed that miyabenol C indeed dramatically inhibited β-secretase activity in both N2aWT and SY5Y cells (Fig. 4A, B). Moreover, miyabenol C markedly inhibited BACE1 acitivity *in vitro*, and its effect was comparable to that of β-secretase inhibitor II *in vitro* (Fig. 4C). Since there is another possibility that miyabenol C inhibits APP β-processing and Aβ generation through promoting α-secretase activity, we also assayed the activity of TACE, a major α-secretase in SY5Y cells treated with miyabenol C. We found that miyabenol C did not affect TACE activity (Fig. 4D), suggesting that miyabenol C does not affect α-secretase activity.

### Miyabenol C treatment reduces sAPPβ and soluble Aβ levels in the brains of APP/PS1 transgenic AD mice

**Figure 5:**
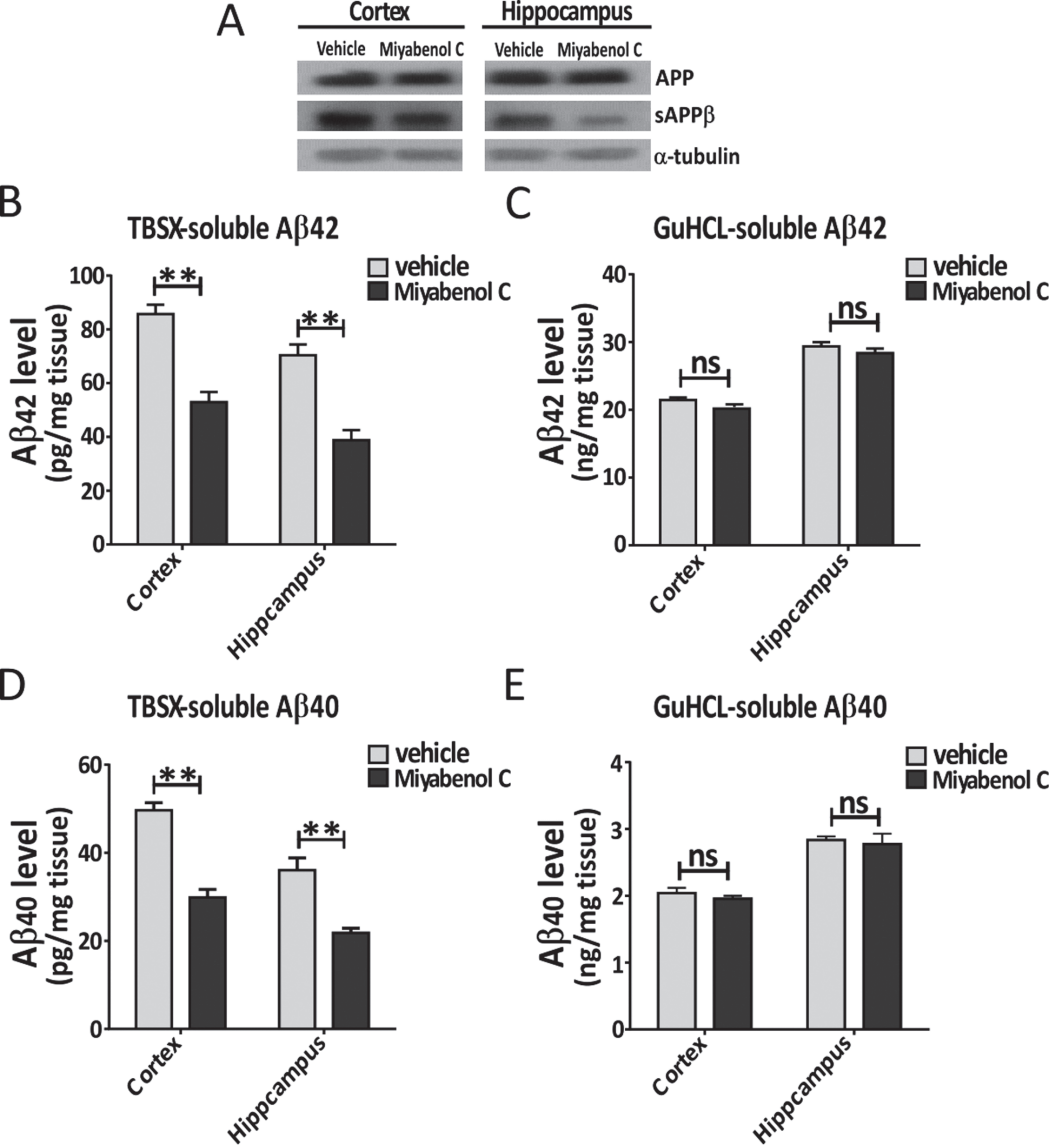
Short term miyabenol C treatment reduces sAPPβ and soluble Aβ levels in APP/PS1 AD mouse brain. APP/PS1 AD mice (12 month old) were treated with miyabenol C (0.6μg/g) or vehicle (45% DMSO in artificial cerebrospinal fluid) through intracerebroventricular injection (4μl) for 72h. (A) Cortex and hippocampus of treated mice were dissected and lysed. Equal protein amounts of lysates were analyzed by Western blot. Levels of TBSX-soluble Aβ42 (B) and Aβ40 (D), as well as TBSX-insoluble (re-dissolved in GuHCl) Aβ42 (C) and Aβ40 (E) in the cortex and hippocampus of treated mice were measured by ELISA assays (Student’s t test, n = 6, ns: *p*>0.05, **: *p*<0.01).

We also examined the *in vivo* inhibitory effects of miyabenol C on β-secretase activity using APP/PS1 transgenic mice, a transgenic mouse model of AD that expresses APP Swedish mutant and PS1 exon-9 deletion mutant. We injected miyabenol C or vehicle control into the lateral ventricle of 12 month-old APP/PS1 mice for a short period of time (72h). Mice were then sacrificed for analyses. We found that mice that received miyabenol C treatment had markedly reduced sAPPβ levels in both cortex and hippocampus when compared with mice treated with vehicle (Fig. 5A). Moreover, miyabenol C treatment significantly reduced levels of Aβ42 and Aβ40 in TBSX-soluble fractions (Fig. 5B, D). However, neither Aβ42 nor Aβ40 levels in the TBSX-insoluble fractions were affected by miyabenol C treatment (Fig. 5C, E).

## Discussion

Natural products provide excellent sources of new therapeutic compounds for disease intervention. Resveratrol and its derivatives are rich in grapes and possess strong anti-oxidant functions. Although resveratrol and its derivatives are considered to have therapeutic potential in AD [37, 38], detailed mechanism underlying their protective effects has yet to be determined.

Miyabenol C is a resveratrol trimer and has been found in plants including various *Vitis* species [39, 40], *Caragana* species [41–44], *Carex* species [45, 46], *Parthenocissus quinquefolia* [47], and *Sophora davidii* [48]. Previous studies have shown that miyabenol C possesses various functions, such as being anti-proliferative and pro-apoptotic in tumor cells [45, 49], inhibiting the activity of protein kinas C [44, 50], antagonizing human 5-hydroxytryptamine (5-HT) receptor [51], having estrogenic activity through binding to estrogen receptor [52, 53], and having ecdysteroid antagonistic activity in *Drosophila* cell bioassays [46]. However, information on the role of miyabenol C in AD is scarce, with only one recent study showing that miyabenol C has marginal effect on inhibiting Aβ fibril formation [39].

In the present study, we isolated miyabenol C from the small-leaf grape (*Vitisthunbergii var*. taiwaniana). Importantly, we found that miyabenol C treatment can dramatically reduce Aβ secretion (Fig. 1) and increase sAPPα release (Fig. 3) in cell culture. Since miyabenol C does not affect total APP levels (Fig. 3B), we studied whether miyabenol C affects α-, β- or γ-secretases. Miyabenol C treatment did not affect the protein level of PS1, a major component of the γ-secretase complex, and γ-secretase-mediated Notch processing for NICD generation, suggesting that miyabenol C does not affect γ-secretase. In addition, miyabenol C treatment did not affect protein levels of two major α-secretases, ADAM10 and ADAM17/TACE, and cell-based TACE activity, implying that miyabenol C does not affect α-secretase. In contrast, although miyabenol C treatment did not affect protein levels of the essential β-secretase BACE1, it reduced cell-based β-secretase activity and *in vitro* BACE1 activity, as well as both sAPPβ and APP β–CTF levels, indicating that miyabenol C reduces Aβ generation through inhibiting β-secretase activity. Consistently, we found that short-term (72h) treatment with miyabenol C in the brain of APP/PS1 mice markedly reduced the levels of both sAPPβ and TBSX-soluble Aβ that represent newly generated portion through β-cleavage, whereas miyabenol C treatment did not affect the levels of TBSX-insoluble Aβ that are from aggregated species (Fig. 5).

Together, our results demonstrate that miyabenol C is a prominent β-secretase inhibitor and can reduce Aβ generation both *in vitro* and *in vivo*. Since β-secretase-mediated APP processing is a rate-limiting step for Aβ generation and inhibition of β-secretase activity is a promising strategy for AD intervention, miyabenol C may be a lead compound for AD drug development.

## References

[1] Hardy JA, Higgins GA (1992) Alzheimer’s disease: the amyloid cascade hypothesis. Science 256: 184–185. 1566067 10.1126/science.1566067

[2] Hardy J, Selkoe DJ (2002) The amyloid hypothesis of Alzheimer’s disease: progress and problems on the road to therapeutics. Science 297: 353–356. 12130773 10.1126/science.1072994

[3] Zheng H, Koo EH (2011) Biology and pathophysiology of the amyloid precursor protein. Mol Neurodegener 6: 27 21527012 10.1186/1750-1326-6-27 PMC3098799

[4] Zhang YW, Thompson R, Zhang H, Xu H (2011) APP processing in Alzheimer’s disease. Mol Brain 4: 3. 21214928 10.1186/1756-6606-4-3 PMC3022812

[5] Hussain I, Powell D, Howlett DR, Tew DG, Meek TD, et al. (1999) Identification of a novel aspartic protease (Asp 2) as beta-secretase. Molecular and cellular neurosciences 14: 419–427. 10656250 10.1006/mcne.1999.0811

[6] Vassar R, Bennett BD, Babu-Khan S, Kahn S, Mendiaz EA, et al. (1999) Beta-secretase cleavage of Alzheimer’s amyloid precursor protein by the transmembrane aspartic protease BACE. Science 286: 735–741. 10531052 10.1126/science.286.5440.735

[7] Lin X, Koelsch G, Wu S, Downs D, Dashti A, et al. (2000) Human aspartic protease memapsin 2 cleaves the beta-secretase site of beta-amyloid precursor protein. Proc Natl Acad Sci U S A 97: 1456–1460. 10677483 10.1073/pnas.97.4.1456 PMC26455

[8] Sinha S, Anderson JP, Barbour R, Basi GS, Caccavello R, et al. (1999) Purification and cloning of amyloid precursor protein beta-secretase from human brain. Nature 402: 537–540. 10591214 10.1038/990114

[9] Yang LB, Lindholm K, Yan R, Citron M, Xia W, et al. (2003) Elevated beta-secretase expression and enzymatic activity detected in sporadic Alzheimer disease. Nat Med 9: 3–4. 12514700 10.1038/nm0103-3

[10] Johnston JA, Liu WW, Todd SA, Coulson DT, Murphy S, et al. (2005) Expression and activity of beta-site amyloid precursor protein cleaving enzyme in Alzheimer’s disease. Biochem Soc Trans 33: 1096–1100. 16246054 10.1042/BST20051096

[11] Li R, Lindholm K, Yang LB, Yue X, Citron M, et al. (2004) Amyloid beta peptide load is correlated with increased beta-secretase activity in sporadic Alzheimer’s disease patients. Proc Natl Acad Sci U S A 101: 3632–3637. 14978286 10.1073/pnas.0205689101 PMC373514

[12] Dominguez D, Tournoy J, Hartmann D, Huth T, Cryns K, et al. (2005) Phenotypic and biochemical analyses of BACE1- and BACE2-deficient mice. J Biol Chem 280: 30797–30806. 15987683 10.1074/jbc.M505249200

[13] Hu X, Hicks CW, He W, Wong P, Macklin WB, et al. (2006) Bace1 modulates myelination in the central and peripheral nervous system. Nat Neurosci 9: 1520–1525. 17099708 10.1038/nn1797

[14] Willem M, Garratt AN, Novak B, Citron M, Kaufmann S, et al. (2006) Control of peripheral nerve myelination by the beta-secretase BACE1. Science 314: 664–666. 16990514 10.1126/science.1132341

[15] Wong HK, Sakurai T, Oyama F, Kaneko K, Wada K, et al. (2005) beta Subunits of voltage-gated sodium channels are novel substrates of beta-site amyloid precursor protein-cleaving enzyme (BACE1) and gamma-secretase. J Biol Chem 280: 23009–23017. 15824102 10.1074/jbc.M414648200

[16] Laird FM, Cai H, Savonenko AV, Farah MH, He K, et al. (2005) BACE1, a major determinant of selective vulnerability of the brain to amyloid-beta amyloidogenesis, is essential for cognitive, emotional, and synaptic functions. J Neurosci 25: 11693–11709. 16354928 10.1523/JNEUROSCI.2766-05.2005 PMC2564291

[17] McConlogue L, Buttini M, Anderson JP, Brigham EF, Chen KS, et al. (2007) Partial reduction of BACE1 has dramatic effects on Alzheimer plaque and synaptic pathology in APP Transgenic Mice. J Biol Chem 282: 26326–26334. 17616527 10.1074/jbc.M611687200

[18] Williams P, Sorribas A, Howes MJ (2011) Natural products as a source of Alzheimer’s drug leads. Nat Prod Rep 28: 48–77. 21072430 10.1039/c0np00027b PMC4917364

[19] Ebrahimi A, Schluesener H (2012) Natural polyphenols against neurodegenerative disorders: potentials and pitfalls. Ageing Res Rev 11: 329–345. 10.1016/j.arr.2012.01.006 22336470

[20] Wang CK, Chen LG, Wen CL, Hou WC, Hung LF, et al. (2010) Neuroprotective activity of Vitis thunbergii var. taiwaniana extracts in vitro and in vivo. J Med Food 13: 170–178. 10.1089/jmf.2009.1162 20136452

[21] Dodson SE, Andersen OM, Karmali V, Fritz JJ, Cheng D, et al. (2008) Loss of LR11/SORLA enhances early pathology in a mouse model of amyloidosis: evidence for a proximal role in Alzheimer’s disease. J Neurosci 28: 12877–12886. 10.1523/JNEUROSCI.4582-08.2008 19036982 PMC2669320

[22] Wang KT, Chen LG, Tseng SH, Huang JS, Hsieh MS, et al. (2011) Anti-inflammatory effects of resveratrol and oligostilbenes from Vitis thunbergii var. taiwaniana against lipopolysaccharide-induced arthritis. J Agric Food Chem 59: 3649–3656. 10.1021/jf104718g 21391605

[23] Liu CC, Kanekiyo T, Xu H, Bu G (2013) Apolipoprotein E and Alzheimer disease: risk, mechanisms and therapy. Nat Rev Neurol 9: 106–118. 23296339 10.1038/nrneurol.2012.263 PMC3726719

[24] Biedler JL, Roffler-Tarlov S, Schachner M, Freedman LS (1978) Multiple neurotransmitter synthesis by human neuroblastoma cell lines and clones. Cancer Res 38: 3751–3757. 29704

[25] Olmsted JB, Carlson K, Klebe R, Ruddle F, Rosenbaum J (1970) Isolation of microtubule protein from cultured mouse neuroblastoma cells. Proc Natl Acad Sci U S A 65: 129–136. 5263744 10.1073/pnas.65.1.129 PMC286201

[26] Thinakaran G, Teplow DB, Siman R, Greenberg B, Sisodia SS (1996) Metabolism of the “Swedish” amyloid precursor protein variant in neuro2a (N2a) cells. Evidence that cleavage at the “beta-secretase” site occurs in the golgi apparatus. J Biol Chem 271: 9390–9397. 8621605 10.1074/jbc.271.16.9390

[27] Zhao Y, Wang Y, Yang J, Wang X, Zhang X, et al. (2012) Sorting nexin 12 interacts with BACE1 and regulates BACE1-mediated APP processing. Mol Neurodegener 7: 30 10.1186/1750-1326-7-30 22709416 PMC3439308

[28] Zhang YW, Liu S, Zhang X, Li WB, Chen Y, et al. (2009) A Functional mouse retroposed gene Rps23r1 reduces Alzheimer’s beta-amyloid levels and tau phosphorylation. Neuron 64: 328–340. 10.1016/j.neuron.2009.08.036 19914182 PMC3846276

[29] Das P, Verbeeck C, Minter L, Chakrabarty P, Felsenstein K, et al. (2012) Transient pharmacologic lowering of Abeta production prior to deposition results in sustained reduction of amyloid plaque pathology. Mol Neurodegener 7: 39 10.1186/1750-1326-7-39 22892055 PMC3477045

[30] Youmans KL, Leung S, Zhang J, Maus E, Baysac K, et al. (2011) Amyloid-beta42 alters apolipoprotein E solubility in brains of mice with five familial AD mutations. J Neurosci Methods 196: 51–59. 10.1016/j.jneumeth.2010.12.025 21219931 PMC3049315

[31] Ono M, Ito Y, Kinjo J, Yahara S, Nohara T, et al. (1995) Four new glycosides of stilbene trimer from Foeniculi fructus (fruit of Foeniculum vulgare MILLER). Chem Pharm Bull 43: 868–871.

[32] Li X, Liu Y, Zheng Q, Yao G, Cheng P, et al. (2013) Ferritin light chain interacts with PEN-2 and affects gamma-secretase activity. Neurosci Lett 548: 90–94. 10.1016/j.neulet.2013.05.018 23685131 PMC3724929

[33] Sorensen EB, Conner SD (2010) gamma-secretase-dependent cleavage initiates notch signaling from the plasma membrane. Traffic 11: 1234–1245. 10.1111/j.1600-0854.2010.01090.x 20573067 PMC2919600

[34] Lammich S, Kojro E, Postina R, Gilbert S, Pfeiffer R, et al. (1999) Constitutive and regulated alpha-secretase cleavage of Alzheimer’s amyloid precursor protein by a disintegrin metalloprotease. Proc Natl Acad Sci U S A 96: 3922–3927. 10097139 10.1073/pnas.96.7.3922 PMC22396

[35] Buxbaum JD, Liu KN, Luo Y, Slack JL, Stocking KL, et al. (1998) Evidence that tumor necrosis factor alpha converting enzyme is involved in regulated alpha-secretase cleavage of the Alzheimer amyloid protein precursor. J Biol Chem 273: 27765–27767. 9774383 10.1074/jbc.273.43.27765

[36] Zhang H, Ma Q, Zhang YW, Xu H (2012) Proteolytic processing of Alzheimer’s beta-amyloid precursor protein. J Neurochem 120 Suppl 1: 9–21. 22122372 10.1111/j.1471-4159.2011.07519.x PMC3254787

[37] Vingtdeux V, Dreses-Werringloer U, Zhao H, Davies P, Marambaud P (2008) Therapeutic potential of resveratrol in Alzheimer’s disease. BMC Neurosci 9 Suppl 2: S6 19090994 10.1186/1471-2202-9-S2-S6 PMC2604890

[38] Baur JA, Sinclair DA (2006) Therapeutic potential of resveratrol: the in vivo evidence. Nat Rev Drug Discov 5: 493–506. 16732220 10.1038/nrd2060

[39] Chaher N, Arraki K, Dillinseger E, Temsamani H, Bernillon S, et al. (2014) Bioactive stilbenes from Vitis vinifera grapevine shoots extracts. J Sci Food Agric 94: 951–954. 10.1002/jsfa.6341 23929536

[40] Lambert C, Richard T, Renouf E, Bisson J, Waffo-Teguo P, et al. (2013) Comparative analyses of stilbenoids in canes of major Vitis vinifera L. cultivars. J Agric Food Chem 61: 11392–11399. 10.1021/jf403716y 24171397

[41] Jin Q, Han XH, Hong SS, Lee C, Choe S, et al. (2012) Antioxidative oligostilbenes from Caragana sinica. Bioorg Med Chem Lett 22: 973–976. 10.1016/j.bmcl.2011.12.012 22209460

[42] Liu HX, Lin WH, Yang JS (2004) Oligomeric stilbenes from the root of Caragana stenophylla. Chem Pharm Bull (Tokyo) 52: 1339–1341. 15516758 10.1248/cpb.52.1339

[43] Chen G, Luo H, Ye J, Hu C (2001) Identification and determination of oligomeric stilbenes in the roots of Caragana species by capillary electrophoresis. Planta Med 67: 665–668. 11582547 10.1055/s-2001-17367

[44] Kulanthaivel P, Janzen WP, Ballas LM, Jiang JB, Hu CQ, et al. (1995) Naturally occurring protein kinase C inhibitors; II. Isolation of oligomeric stilbenes from Caragana sinica. Planta Med 61: 41–44. 7700990 10.1055/s-2006-957996

[45] Gonzalez-Sarrias A, Gromek S, Niesen D, Seeram NP, Henry GE (2011) Resveratrol oligomers isolated from Carex species inhibit growth of human colon tumorigenic cells mediated by cell cycle arrest. J Agric Food Chem 59: 8632–8638. 10.1021/jf201561e 21761862

[46] Meng Y, Bourne PC, Whiting P, Sik V, Dinan L (2001) Identification and ecdysteroid antagonist activity of three oligostilbenes from the seeds of Carex pendula (Cyperaceae). Phytochemistry 57: 393–400. 11393519 10.1016/s0031-9422(01)00061-9

[47] Yang JB, Wang AG, Ji TF, Su YL (2014) Two new oligostilbenes from the stem of Parthenocissus quinquefolia. J Asian Nat Prod Res 16: 275–280. 10.1080/10286020.2013.877451 24456249

[48] Tanaka T, Ito T, Iinuma M, Ohyama M, Ichise M, et al. (2000) Stilbene oligomers in roots of Sophora davidii. Phytochemistry 53: 1009–1014. 10820822 10.1016/s0031-9422(00)00016-9

[49] Barjot C, Tournaire M, Castagnino C, Vigor C, Vercauteren J, et al. (2007) Evaluation of antitumor effects of two vine stalk oligomers of resveratrol on a panel of lymphoid and myeloid cell lines: comparison with resveratrol. Life Sci 81: 1565–1574. 18001803 10.1016/j.lfs.2007.08.047

[50] Xu G, Zhang LP, Chen LF, Hu CQ (1994) [Inhibition of protein kinase C by stilbenoids]. Yao Xue Xue Bao 29: 818–822. 7863783

[51] Kim DH, Kim SH, Kim HJ, Jin C, Chung KC, et al. (2010) Stilbene derivatives as human 5-HT(6) receptor antagonists from the root of Caragana sinica. Biol Pharm Bull 33: 2024–2028. 21139245 10.1248/bpb.33.2024

[52] Tian CY, Gao HD, Zhang YG, Xu G, Hu CQ, et al. (2003) [The binding sites of estrogen receptor for miyabenol C and kobophenol A]. Sheng Wu Hua Xue Yu Sheng Wu Wu Li Xue Bao (Shanghai) 35: 77–81. 12518232

[53] Tian CY, Hu CQ, Xu G, Song HY (2002) Assessment of estrogenic activity of natural compounds using improved E-screen assay. Acta Pharmacol Sin 23: 572–576. 12060535

